# Comparison of different pupil dilatation methods for phacoemulsification in eyes with a small pupil

**DOI:** 10.1186/s12886-022-02402-1

**Published:** 2022-04-18

**Authors:** Jin Da Wang, Jing Shang Zhang, Meng Li, Ying Yan Mao, Yusufu Mayinuer, Xiu Hua Wan

**Affiliations:** Beijing Tongren Eye Center, Beijing Tongren Hospital of Capital Medical University, Beijing Key Laboratory of Ophthalmology and Visual Sciences, Beijing, 100005 China

**Keywords:** Cataract, Phacoemulsification, Small pupil, Pupil dilatation, Pupil expander

## Abstract

**Purpose:**

To compare 6 methods for intraoperative pupil dilatation in eyes with insufficient pupil size during phacoemulsification.

**Methods:**

This was a prospective case–control study. 99 microcoria cataract patients (120 eyes) were collected and were divided into 6 groups(20 eyes each group), and their pupils were dilated by bimanual stretching pupil (group I), pupil radial cut open(group II), mechanical pupil dilatation with iris-retractor hooks (group III), OASIS iris expander (group IV), and Malyguin-ring (Microsurgical company, America) (group V), B-HEX Pupil Expander (Med Invent Devics, India)(group VI),respectively. 3.0 mm clear corneal incision were used in phacoemulsification. All cases were followed up at 1 week and 1, 3, 6 months after the surgery. The best corrected visual acuity (BCVA), intraocular pressure(IOP), corneal endothelium cell density(ECD), pupil diameter(PD) of before and after surgery were compared.

**Results:**

One same doctor finished all cataract surgeries successfully. The eyes’ condition before surgery and at 6 months after surgery were compared. There were no significant statistical differences for the conditions of the eyes before surgery among six groups. The ECDs were better at 6 months postoperatively in group III and V, median values: 2114/mm^2^, 1961/mm^2^. PD was largest in group II (median value: 5.5 mm), which was significantly larger than other groups (P_adjusted_ < 0.05).

**Conclusions:**

All 6 methods used in this study were effective for the mechanical dilatation of small pupils and didn’t affect the postoperative visual acuity and intraocular pressure in microcoria cataract phacoemulsification. Iris-retractor hooks and the Malyugin Ring can reduce intraoperative corneal endothelium cell loss. Postoperative PD is larger when the iris was cut open radially.

## Introduction

In 2015, cataract was identified as the leading cause of moderate or severe vision impairment globally [1]. The small, poorly dilated pupil is one of the most common difficulties faced by cataract surgeons [2]. Study estimated that small pupil cataracts accounted for about 11 percent of all cataract operations [2]. There are many reasons for the failure of the pupil dilation during cataract surgery. Common small pupil cataracts are pupil adhesions due to a variety of causes, including prior trauma or surgery, uveitis, and chronic mycotic therapy for glaucoma. In addition, some eye diseases and systemic diseases can also lead to the failure of the pupil dilation during the operation, such as senile pupillary sphincter sclerosis, pseudo cystectomy syndrome [3], intraoperative iris relaxation syndrome [4], iris cleft, diabetes, etc. Small pupil makes the surgery more difficult to perform and causes more postoperative damage response. The incidence of complications such as capsular rupture and vitreous loss increases remarkably. As the small pupil seriously affects the cataract surgery, several methods are adopted to deal with it, such as, sphincterectomy, iris hooks, stetch pupilloplasty techniques and pupil-dilator rings. In this study, our aim is to compare 6 methods for intraoperative pupil dilatation in eyes with insufficient pupil size during phacoemulsification.

## Methods

### Subjects

A total of 99 cataract patients (120 eyes) with small pupil who received phacoemulsification with a 3.0 mm clear cornea incision between January 2015 and October 2019 in Beijing Tongren Hospital were included.

## Examinations

All patients underwent routine preoperative examinations and regular postoperative follow-up as previously reported [5]. All patients’ nuclear color graded based on the Lens Opacities Classification System III criteria [6]. The pupil diameter (PD) was measured by pupil gauge,The anterior chamber depth(ACD) was measured with A scan ultrasound.

## Surgical procedure

**Fig. 1:**
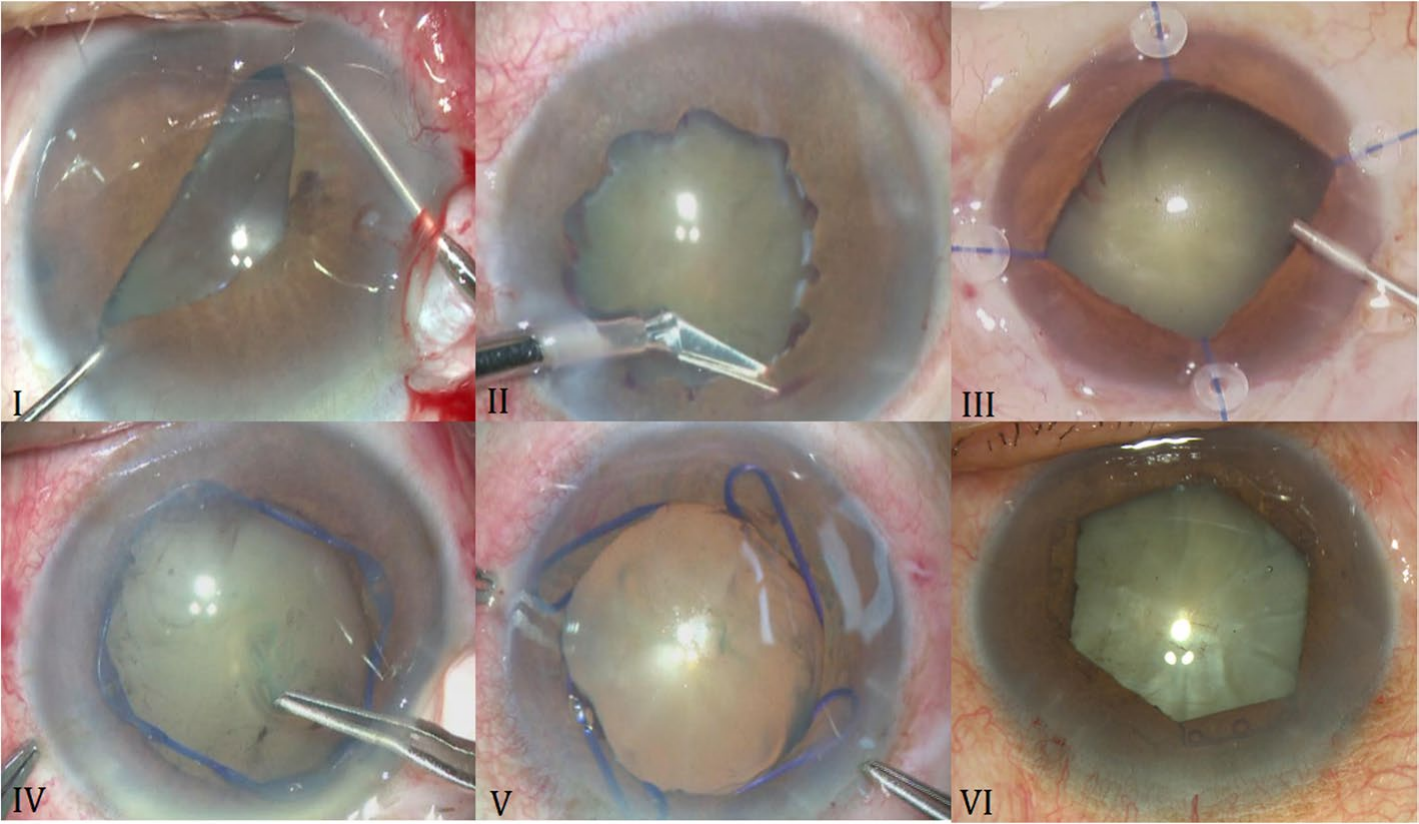
The six pupil dialated methods: group I: manual stretch, group II: iris radial cut open, group III:iris hooks, group IV: OASIS iris expander, group V: malyugin ring,group VI: B-HEX pupil expander

All cataract phacoemulsification and IOL implantations were finished successfully by same surgeon (WXH) as previously reported [5]. The small pupils were dilated by six different methods showed in Fig. 1: bimanual stretching pupil (group I), pupil radial cut open (group II), mechanical pupil dilatation with iris-retractor hooks(group III), OASIS iris expander(group IV), and Malyguin-ring (Microsurgical company, America)(group V), B-HEX Pupil Expander (Med Invent Devics, India)(group VI).

## Statistical analysis

Sample size estimation and statistical analysis was performed using the open source R program (https://www.r-project.org/, version4.2.0). Frequency and percentage was used for basic statistical description of categorical variables. Shapiro-Wilc test was used for normality test of continuous variables, mean values and standardized deviation was used for basic statistical description of normally distributed continuous variables, otherwise median values and inter-quartile range was used.

Linear mixed effects model was used to adjust the effect of age and gender, then comparison of outcomes among 6 groups was performed.

Post-hoc test was done when there was statistical difference in outcomes among 6 groups, and *P* value was adjusted according to Bonferroni criteria. Significance level was set to be 0.05, two tailed.

## Results

A total of 120 eyes of 99 cataract patients (45 males and 54 females) with small pupil were included in this study. The normality test of continuous variables was shown in Table 1. The demographic characteristics and data on the eyes of the patients are summarized in Table 2.

**Table 1:** Normality test results

Variables	Statistics (W)	*p*
BCVA POST	0.833	< 0.001
IOP POST	0.984	0.200
ECD POST	0.970	0.010
PD POST	0.947	< 0.001
Age	0.992	0.735
ACD PRE	0.963	0.631
BCVA PRE	0.898	< 0.001
IOP PRE	0.991	0.675
ECD PRE	0.995	0.965
PD PRE	0.913	0.974

*PRE* Preoperative, *POST* Postoperative

**Table 2:** The demographic characteristics of the patients and data of the eyes before surgery of the 6 groups

Group	I	II	III	IV	V	VI
Method	manual stretch	iris cut	iris hook	OASIS	Malyugin Ring	B-HEX
Male	6	8	9	8	7	7
Female	10	9	7	10	9	9
Eyes	20	20	20	20	20	20
Age(years)	66.5 ± 12.1(29–82)	58.2 ± 10.4(44–71)	57.6 ± 11.3(45–66)	64.9 ± 12(43–81)	66.6 ± 9(52–79)	59.8 ± 9(47–67)
BCVA PRE(LogMar)	1.0(0.2–1.7)	0.6(0.5–0.9)	0.9(0.6–1.0)	1.3(0.7–2.0)	2.0(1.0–2.7)	1.3(0.7–1.7)
IOP PRE(mmHg)	14.7 ± 4.6(9–22)	20.14 ± 4.67(14–24)	15.2 ± 3.7(13–19)	15.7 ± 5.7(8–24)	16.1 ± 5.1(9–26)	13.25 ± 1.9(12–16)
ECD PRE(/mm^2^)	2490 ± 498(1523–3723)	2644 ± 219(2337–3010)	2529 ± 613(2020–3255)	2424 ± 640(1186–3271)	2620 ± 989(1768–3570)	2182 ± 752(1718–3051)
PD PRE(mm)	2.05 ± 0.60(1.5–3.5)	1.86 ± 0.85(0.5–2.5)	1.96 ± 0.65(1–2.5)	1.94 ± 0.5(1–4)	2.23 ± 0.85(1–3.5)	2.25 ± 0.65(1.5–3)
ACD PRE	2.85 ± 0.16(2.12–3.5)	2.32 ± 0.46(1.91–2.93)	2.68 ± 0.41(2.19–2.99)	2.74 ± 0.56(2.01–3.47)	2.32 ± 0.49(1.67–3.5)	2.7 ± 0.31(2.23–2.85)
Uveitis	8	10	11	8	10	10
Glaucoma	10	10	9	12	9	10
Others	2	0	0	0	1	0

*PRE* Preoperative

This study first tested the normality of continuous variables, preoperative BCVA, postoperative BCVA, postoperative ECD, and postoperative PD were not normally distributed.

According to normality test (Table 1), we used median values and inter-quartile range to make basic statistical description of preoperative BCVA, postoperative BCVA, postoperative ECD, and postoperative PD, while for other continuous variables, mean values and standardized deviations were used, as shown in Table 2.

This study was designed as a multi-group parallel control, and the difference test was used to compare the groups. ECD was one of the main outcome index of this study. According to the preliminary experimental results of the average ECD of the six groups, at least 8 samples were calculated for each group. So we collected 20 cases in each group that should meeted the statistical analysis requirements.

All patients had nuclear opalescence (NO) 3 or nuclear color (NC) 3 and NO4 or NC4 based on the Lens Opacities Classification System III criteria [6]. There were no statistically significant differences in age among the 6 study groups and there were also no statistically significant differences in IOP, ECD, ACD, pupil diameter before suegery among the 6 groups(*P* > 0.05). All surgeries were performed successfully without any intraoperative complications. The power of implanted IOL ranged between 19.5 and 30.00D with an average of 24.44 ± 4.30 D.

### The BCVA and IOP

The BCVA of all patients were improved after surgery. The pairwise comparison of BCVA was different between groups, there was significance difference in postoperative BCVA among 6 groups (Table 3), patients in the IV group showed the best BCVA (median value: 0). The IOP values of the 6 groups were pairwise compared, and there was no significant difference in IOP values among the groups.

**Table 3:** Postoperative distribution of indicators in 6 groups

VARIABLE	manual_stretch	iris_cut	iris_hook	OASIS	Malyugin_Ring	B_HEX	*P*
BCVA post	0.3(0.2–0.4)	0.1(0–0.2)	0.2(0.1–0.3)	0(-0.1–0.1)	0.2(0.1–0.5)	0.1(0–0.2)	< 0.001
IOP post	14.1 ± 6.4(8–25)	17 ± 5.06(12–26)	13.1 ± 4.21(12–21)	12.67 ± 6.35(12–15)	12.4 ± 2.2(8–15)	14.7 ± 4(11–19)	0.062
ECD post	1452 (1159–1622)	1773 (1558–2075)	2114 (1795–2281)	1328 (904–1830)	1961 (1665–2352)	1725 (1028–2306)	< 0.001
PD post	4(3–4.5)	5.50(5.-6)	3.5(2.5–4)	5(3–6)	4(3–5)	2.5(2–3)	< 0.001

The results in Table 3 showed that postoperative BCVA between the six groups was statistically different in at least two groups, as were postoperative ECD and postoperative PD. There was no statistical difference in postoperative IOP among the 6 groups

### The corneal endothelium cell density

The corneal endothelium cell density decreased in all patients after surgery. The pairwise comparison of corneal endothelium cell density showed differences between groups (Table 4). The corneal endothelium cell density in group III and V were better (median values: 2114/mm^2^, 1961/mm2) than that in group I, II, IVand VI (1452/mm^2^, 1773/mm^2^, 1328/mm^2^,1725/mm^2^), as shown in Table 3.

**Table 4:** Comparison of ECD values between 6 groups after operation

Comparison between 2 groups		*p*	P.adj
I	II	0.301	0.900
I	III	0.000	0.004
I	IV	0.033	0.160
I	V	0.000	0.004
I	VI	0.102	0.410
II	III	0.067	0.680
II	IV	0.067	0.680
II	V	0.201	1
II	VI	0.779	1
III	IV	0.000	< 0.001
III	V	0.355	0.900
III	VI	0.134	1
IV	V	0.000	< 0.001
IV	VI	0.738	0.900
V	VI	0.174	1

p.adj stands for p value that adjusted according to Bonferroni criteria

*I* manual stretch, *II* iris cut, *III* iris hook, *IV* OASIS, *V* Malyugin Ring, *VI* B-HEX

### The pupil diameter

The pupil diameter of all eyes were larger after surgery. The pairwise comparison of pupil diameter showed differences between groups (Table 5). The pupil diameter was largest in group II (median value:5.5 mm), as shown in Table 3.

**Table 5:** Comparison of PD values between 6 groups after operation

Comparison between 2 groups		*p*	P.adj
I	II	< 0.001	< 0.001
I	III	0.947	1
I	IV	0.003	0.027
I	V	0.758	1
I	VI	0.001	0.013
II	III	< 0.001	< 0.001
II	IV	< 0.001	0.006
II	V	< 0.001	< 0.001
II	VI	< 0.001	< 0.001
III	IV	0.003	0.027
III	V	0.718	1
III	VI	< 0.001	< 0.001
IV	V	0.003	0.027
IV	VI	< 0.001	< 0.001
V	VI	0.014	0.057

p.adj stands for *p* value that adjusted according to Bonferroni criteria

*I* manual stretch, *II* iris cut, *III* iris hook, *IV* OASIS, *V* Malyugin Ring, *VI* B-HEX

## Discussion

A well-dilated pupil is one of the requirements for safe and successful phacoemulsification surgery. Therefore, it is important to find a method for proper pupil dilation for an cataract doctor when faced a small pupil cataract surgery. Techniques for intraoperative mechanical dilatation of the pupil must be effective, safe, quick, practical, and economical. In this study, we compared 6 methods for pupil dilation.

The results of this study showed that all these methods could achieve proper pupil dilation and improve the safety of the surgery, without affecting intraocular pressure. Although BCVA differed between the six groups, BCVA was mainly related to the state of fundus of patients under the condition of good refractive media, and should have little relationship with pupil dilation method.

The pupil maintained dilated better in group III-VI than in group I-II because the intraocular instrument can hold the iris. In group I and II, the pupils may resume to a smaller size, making the surgery more difficult to perform, while the costs for patients in group III-VI were higher with the use of intraocular instrument.

Procedures of surgeries in group III-VI were more complicated because of the use of intraocular instrument. More incisions were required to use the iris hooks in group III [7]. Compared with the iris hooks, the advantage of the Malyugin Ring, the OASIS pupil dilator and the B-HEX pupil expander was that there is no need for another incision. The OASIS iris expander in group IV was thicker and harder than Malyugin Ring in group V and the B-HEX pupil expander in group VI, and thus its manipulation would be more complicated. The B-HEX pupil expander was easy to fall off from the pupil sometimes in practice. We thought it maybe because the B-HEX pupil expander was very thin and with weak elasticity. The Malyugin Ring was more reasonably designed and much simpler to operate in our practice.

Before the Malyugin Ring, OASIS iris expander and the B-HEX pupil expander were used, there had been other pupil dilators, such as Morcher pupillary dilator [8, 9], Graether pupillary dilator [10, 11], Perfect the pupil dilator [11, 12], and Siepser pupil dilation etc.. These pupil dilators were designed to be implanted through the main incision in cataract phacoemulsification, fixed at the pupil margin to dilate the pupil, and removed from the main incision at the end of the surgery. Their common disadvantage was that the volume was large and some materials were hard. It was time-consuming to implant them into the eyes and remove them out. The doctors would need a much longer time to study using them and tissue damage is relatively severe. Hence, they were not widely applied. The lightweight, square linear design of Malyugin ring [13–15] and OASIS iris expander cleverly avoided these drawbacks. Additionally, they both have an independent disposable micro implantation-removal system, and thus their volumes are quite small when implanted. The Malyugin ring is relatively thin and elastic, and each corner has a circle for the fixation of iris. The OASIS iris expander is relatively thick and hard, and each corner had a triangular bracket for the fixation of iris. The B-HEX pupil expander [16] is designed six side,not the same to the Malyugin ring and the OASIS iris expander. The B-HEX pupil expander is more softer than the Malyugin ring and don’t need a disposable injector which increases the cost of production.

Corneal endothelium damage was severer in group I, II, IVandVI than in group III and V. There were no statistically significant differences in the corneal endothelium cell density and ACD among the 6 groups before surgery, so we think it might be attributed to less intraocular operations in group III and V [17].

The pupil diameter was largest in group II, which may affect the visual quality. The iris was radially cut open in group II, which will inevitably injure pupillary sphincter, thereby disabling the pupil contraction. The pupillary sphincter was pulled in an evenly dispersed fashion in the other five groups, which could minimize the damage of pupil sphincter in the process of pupil dilation, thereby effectively keeping the function of pupil sphincter intact and facilitating the recovery of postoperative visual quality.

This study compared the six pupil dilation methods for phacoemulsification in eyes with small pupil. Each method has its own advantages and disadvantages. Doctors should choose the suited method for each individual case. The limitation of this study is that the number of cases was limited, and the sample size used for statistical analysis was small.

## Conclusions

All 6 pupil dilation methods used in this study were effective for the mechanical dilatation of small pupils. Iris-retractor hooks and the Malyugin Ring can reduce intraoperative corneal endothelium cell loss. Postoperative PD is larger when the iris was cut open radially.

## Data Availability

The datasets of the current study are available from the corresponding author on reasonable request.
